# Cognitive load influences oculomotor behavior in natural scenes

**DOI:** 10.1038/s41598-021-91845-5

**Published:** 2021-06-11

**Authors:** Kerri Walter, Peter Bex

**Affiliations:** Psychology Department, Northeastern University, Boston, 02115 USA

**Keywords:** Neuroscience, Psychology

## Abstract

Cognitive neuroscience researchers have identified relationships between cognitive load and eye movement behavior that are consistent with oculomotor biomarkers for neurological disorders. We develop an adaptive visual search paradigm that manipulates task difficulty and examine the effect of cognitive load on oculomotor behavior in healthy young adults. Participants (N = 30) free-viewed a sequence of 100 natural scenes for 10 s each, while their eye movements were recorded. After each image, participants completed a 4 alternative forced choice task in which they selected a target object from one of the previously viewed scenes, among 3 distracters of the same object type but from alternate scenes. Following two correct responses, the target object was selected from an image increasingly farther back (N-back) in the image stream; following an incorrect response, N decreased by 1. N-back thus quantifies and individualizes cognitive load. The results show that response latencies increased as N-back increased, and pupil diameter increased with N-back, before decreasing at very high N-back. These findings are consistent with previous studies and confirm that this paradigm was successful in actively engaging working memory, and successfully adapts task difficulty to individual subject’s skill levels. We hypothesized that oculomotor behavior would covary with cognitive load. We found that as cognitive load increased, there was a significant decrease in the number of fixations and saccades. Furthermore, the total duration of saccades decreased with the number of events, while the total duration of fixations remained constant, suggesting that as cognitive load increased, subjects made fewer, longer fixations. These results suggest that cognitive load can be tracked with an adaptive visual search task, and that oculomotor strategies are affected as a result of greater cognitive demand in healthy adults.

## Introduction

### Where do we look?

The human visual system only allows for high-resolution visual information to be encoded from the fovea (the central ~ 2° of vision). As a result, to estimate the contents of a scene, we move our eyes rapidly around a scene (*saccades*) in order to focus our central vision on multiple discrete areas (*fixations*) (for review, see^1,2^).

Human vision is reliant on eye movements, however there is still relative debate about what determines where observers will look when told to view a scene. It is well documented that subjects adopt different viewing strategies when performing different tasks, as the way someone looks around a scene is dependent on the current task they are trying to accomplish^3,4^. However, it is still unclear how subjects decide where to look when they are given no task or instruction, otherwise known as “free-view”. During free-view, fixation locations may vary significantly from subject to subject^5^. Because the ways different individuals view a scene are idiosyncratic, it is unclear what exactly guides eye movements during free-view.

Two main approaches have attempted to explain what guides eye movements during free-view: salience and meaning. Evidence suggests that fixation locations may be driven by areas of higher salience^6–9^, while opposing evidence suggests fixation locations are driven by areas of higher semantic meaning^10–16^. The salience approach is based on bottom-up processes, stating that fixations are guided by image features that contrast their surroundings, while the meaning approach is based on top-down processes, stating that fixations are guided by prior experience. Additionally, some evidence suggests that fixation durations may be guided by peripheral content and image features^17,18^. Yarbus’ original study demonstrates that participants will have different scan-paths for the same image, even while performing the same task, suggesting that low level information is not sufficient to predict human gaze^4^. Recently, deep learning models of gaze-guidance have trained convolution neural networks on the gaze patterns of human subjects and have demonstrated greater performance than salience or meaning models alone^19^. These approaches therefore indirectly incorporate both feed-forward scene statistics with the use of high-level image meaning that guided the fixations of observers who supplied the training eye movements.

Eye movements have proven to be useful diagnostic tools and biomarkers for cognitive functioning. For example, children with reading difficulties exhibit atypical oculomotor behaviors while reading^20^, and children with autism spectrum disorder exhibit subtle atypical oculomotor behaviors when processing language and social information^21,22^, as well as exhibiting a center bias on images, demonstrating reduced saliency for social-gaze related locations, and prioritizing saliency for pixel-specific locations rather than saliency for overall semantic knowledge^22^. Eye movements can also serve as screening methods for degenerative diseases, such as Alzheimer’s, as saccades and smooth pursuit become slowed and less accurate, and viewing strategies become erratic and seemingly random^23^.

### Cognitive load

Cognitive load refers to the amount of active effort being invoked by working memory^24^. N-back tasks have been widely utilized to measure working memory function, therefore cognitive load can be manipulated with the use of an N-back task. An N-back task presents participants with visual or auditory information and asks the participant to remember that information a specified number (N) of trials later^25^. Generally, as N-back increases, response latencies increase and response accuracies decrease^26–28^. Increasing the demands of an N-back task has also been shown to activate various areas of the brain associated with working memory^26–30^.

Some studies have demonstrated that eyetracking technology can be used to measure cognitive load, with features such as pupilometry: pupil diameter has been shown to increase in response to increasing levels of cognitive load^31–34^. Different aspects of oculomotor properties (fixation number and duration; saccade length, angle, and velocity; pupil dilation; blink rate and velocity) have been linked to cognitive load^35–37^, and combinations of these features have been proposed as a model for measuring cognitive load^36^.

### Cognitive load vs perceptual load

Top-down processing can be affected by an increase in cognitive load, but not perceptual load^38^. Perceptual load refers to the amount of visual information being presented, and is related to the levels of clutter, distractors, or edges within a scene. Perceptual load is therefore distinct from cognitive load, which refers to the amount of information being processed in the brain, and is related to working memory^38^. Belke et al.^38^ demonstrated that tasks which required semantic knowledge, such as matching a written word with its line drawing, were influenced by the presence of a competitor object, (an object similar in semantic meaning), when assigned an additional working memory task (increased cognitive load), but were not influenced when the number of objects on screen increased (increased perceptual load). This distinction ensures that we can present a variety of natural images to subjects while strictly manipulating cognitive load, with perceptual load remaining unaffected.

To summarize: if visual search strategies are guided by top-down processing, and increasing cognitive load disrupts top-down processes, then increasing working-memory demands (which we will do with an N-back task) should alter a participant’s visual search strategy. We are interested in seeing if this increased cognitive demand affects a subject’s oculomotor strategies, such as the number and duration of fixations and saccades. Similarly, do subjects who excel with this task, (subjects who can hold a higher number of scenes in working memory, or subjects with higher cognitive load capacities), utilize different oculomotor strategies than subjects who struggle with this task (subjects with low cognitive load capacities)? Do certain oculomotor strategies predict accuracy on this task? We modify an N-back paradigm for visual search in natural scenes and implement an adaptive procedure to maintain constant cognitive load, given large individual differences in visual search performance. The task allows for the analysis of oculomotor behaviors under varying levels of cognitive load.

Similar studies demonstrate a close relationship between attention, cognitive function, and the deployment of eye movements^26,29,39,40^. We therefore hypothesize that changes in attention demand and cognitive load should lead to reliable changes in oculomotor behavior. We also hypothesize that individual differences in performance on a demanding cognitive task should be associated with differences in patterns of oculomotor behavior. In this study, we manipulate cognitive load in a healthy population of young adults and measure eye movement behavior as they perform a demanding visual search task in natural scenes. If an overwhelming increase in cognitive load in healthy controls is similar to cognitive impairment, we would expect oculomotor behaviors at high N-backs to replicate those of cognitively impaired individuals. In general, the average number of oculomotor behaviors while scanning the environment decreases with cognitive impairment^21,41,42^. In an alternate hypothesis, perhaps success in this task is dependent on making many rapid eye movements in order to visually capture as much of the scene as possible. In this case, we would expect more oculomotor behaviors as N-back increases, as subjects must be consistently successful in the task in order to achieve higher N-backs. In this study, we examine if oculomotor behavior, regardless of scene context, can explain some of the differences between how individual subjects view a scene. We propose an adaptive N-back task that allows for the comparison of oculomotor behaviors under varying levels of cognitive load.

## Methods

### Apparatus

**Figure 1:**
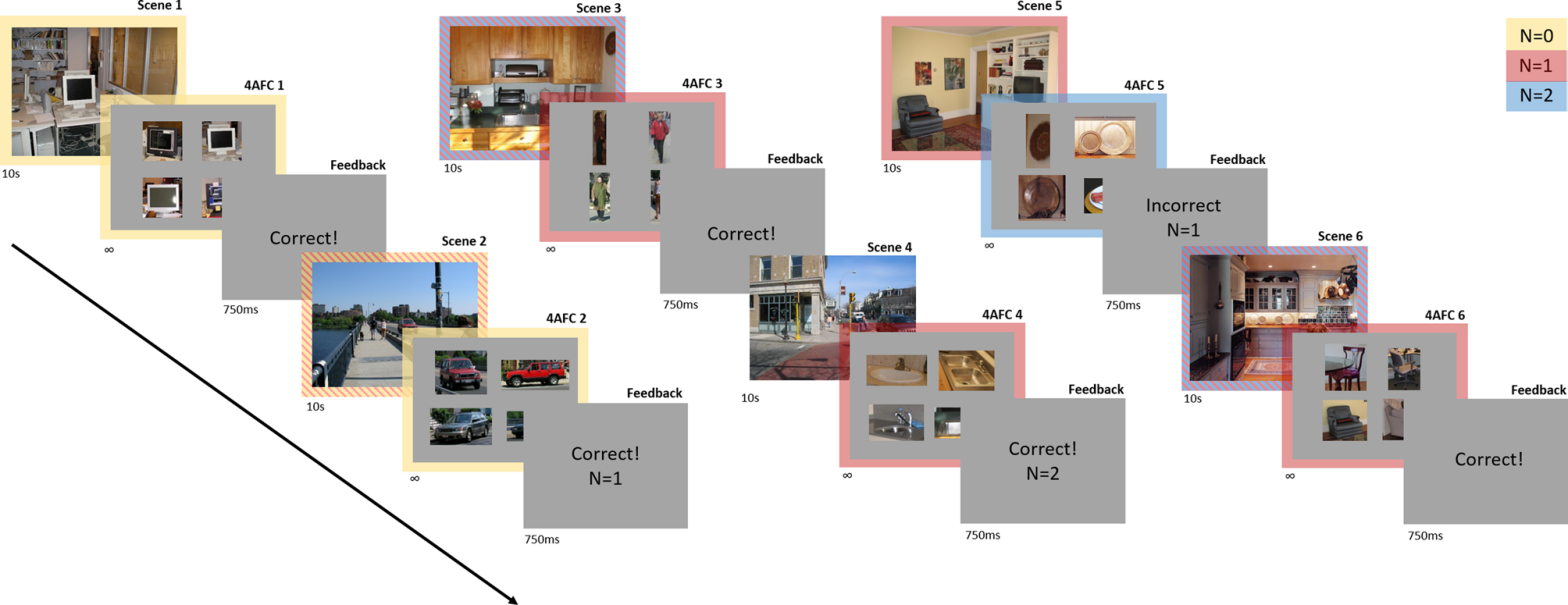
Illustrations of N-back Visual Search. (**A**) Example of the order of events within the experiment. Subjects viewed a sequence of scenes for 10 s each, were given unlimited time to complete a 4 Alternate Forced Choice task (4AFC) of similar objects, one of which was from a scene viewed *N* images back, then received feedback for 750 ms before viewing a new scene. Scenes highlighted in yellow represent those which were used in an N = 0 4AFC, scenes highlighted in red represent those which were used in an N = 1 4AFC, and scenes highlighted in blue represent those which were used in an N = 2 4AFC. Because of the nature of our adaptive task, some scenes were used in multiple 4AFCs (highlighted with two cross-hatched colors), and some images were not used in any 4AFCs (no highlighted color). The same colors are used in the legend to depict N-back for each 4AFC task. All images used are from the LabelMe database^45^, made publicly available to the research community without restrictions.

Stimuli were presented on a 60 cm × 34 cm BenQ XL2720Z LCD monitor (BenQ Corporation, Taipei, Taiwan) set to a screen resolution of 1920 × 1080 pixels at 120 Hz and run using a Dell Optiplex 9020 desktop computer (Dell Inc. Round Rock, TX) with a Quadro K420 graphics card. The experiment was programmed and run using MATLAB (The MathWorks, Inc., Natick, MA) and the Psychophysics Toolbox Version 3^43^. Observers were seated 63 cm from the monitor with head stabilization secured via chinrest. Eye movements were recorded using an SR Research Eyelink 1000 (SR Research Ltd. Mississauga, Ontario, Canada) and the MATLAB Eyelink Toolbox^44^. The sampling rate was set to 1000 Hz (note that sampling rate was set to 250 for one subject due to experimenter error, however this did not impede data collection or analysis). Figure 1 was created using PowerPoint 2013 (https://www.microsoft.com/en-us/microsoft-365/previous-versions/microsoft-powerpoint-2013), all other figures were created using Matlab Version 9.6 (https://www.mathworks.com/products/matlab.html).

### Participants

In total, 33 naïve subjects (7 male, 26 female) with self-reported normal or corrected vision from the Northeastern undergraduate population participated in this study. 3 subjects were excluded due to program crashes (N = 2) or Eyelink calibration issues (N = 1). Subjects were excluded as soon as issues arose, and data collection continued until 30 subjects with usable data were collected (7 male, 23 female). Subjects received course credit as compensation for their time. All subjects read and signed an informed consent form approved by the University Ethics Board before the experiment began, the experimental procedure was approved by the institutional review board at Northeastern University, and the experiment was performed in accordance with the tenets of the Declaration of Helsinki.

### Images

In total, 100 images (50 indoor, 50 outdoor), were selected from the LabelMe database^45^. The database, comprised 75,353 total images at the time of selection, was filtered down as a result of the steps listed in Table 1. All images were landscape oriented and were in color.

**Table 1:** Steps taken to filter through the LabelMe database.

Images removed	Images remaining
< 75% of image surface labeled	11,822
< 15 unique objects	2186
< 1000 × 1000 pixel resolution	1629
Portrait images	1523
≥ 25% of unique objects are parts of larger objects	1364
≥ 50% of image is taken up by a single object	1311
< 15 unique objects (excluding broad scenery objects)	975

List of filters applied, and number of images remaining after filtering, that lead to the unbiased selection of 100 experimental images.

From these remaining 975 images there were 76 indoor and 899 outdoor scenes, from which we hand selected 50 indoor and 50 outdoor images. Images were manually removed based on criteria similar to above: we removed images with objects taking up a large portion of the frame, blurry images, images with few distinct objects, etc. We also avoided including images that were taken of the same setting at different angles, to ensure no identical objects were overlapping in the database. We sought to ensure that the image database used for this experiment was varied, but also that each image had enough common, unique objects to satisfy the decision task. The mean luminance value of our images was 122.67rgb steps with a standard deviation of 14.97rgb steps, and the images were presented in discrete random order to all subjects.

### Procedure

Participants were shown a short schematic of the instructions (in the form of a PowerPoint presentation) before the experiment began. Subjects were asked if they understood the task before the start of the experiment. All subjects reported yes, and none reported that they struggled with the task due to misunderstanding the instructions. Participants were shown an image for 10 s and were instructed to view the scene freely. After 10 s, the image was removed and replaced with four small snapshots from different scenes, each centered on objects with the same label from the LabelMe database. One of these snapshots was from the image the participant had previously viewed, and the goal was to identify the corresponding object by clicking a mouse cursor on it. For example, a forced choice task could be of four different lamps, with one of the lamps from the target scene, and the other three from other scenes within the experiment, without replacement. Participants received immediate feedback on their answer. Whenever a subject answered two trials correctly in a row, they received a prompt that read “Now look for objects from the image (N) back”. N would change depending on subject’s performance. N started at zero, meaning the choice task was referring to the image immediately preceding it. Every time a subject answered two trials in a row correctly, N was increased by one. If at any point a subject answered incorrectly, N was decreased by one (Fig. 1).

The experiment was composed of 100 trials across 4 blocks (25 trials per block). A standard Eyelink 9-point eye tracker calibration task was completed before the start of each block. Images were presented in random order for each participant. There was a mandatory break between blocks, and participants were instructed to tell the experimenter when they were ready to continue. Participants were told that they did not have to remember the previous image stream during a break, as N was reset to zero at the start of each new block.

All images were scaled to be approximately the same size (1280 × 960 pixels) when presented in the experiment. Images were rescaled according to their largest dimension in order to maintain their original aspect ratio. The forced choice task was comprised of objects taken from the 100 images used in the dataset. For each trial, one object was randomly chosen from the list of labeled objects in the LabelMe file for each image. The full database was scanned for matches of that object label. If the object did not reoccur at least 3 times within the dataset, a different object was chosen. Three objects with the same label were chosen at random and used as distracters alongside the target object in the forced choice task. Only one object was sampled from each image at a time. Only objects larger than 100 × 100 pixels were used to prevent excessive magnification in the alternative choice display. Objects were taken from a rectangular section of the original image, with a surrounding 10% of the object’s dimensions included. This was done to provide a small amount of image context for each object. In pilot studies, we grabbed only the object with no background context for the alternative choice display, however, this proved to be too difficult for subjects to complete. The objects were scaled to be approximately the same size as each other (maximum dimension of 300 pixels), while maintaining their original aspect ratios, but different from their size in the original scene.

Lighting conditions in the experimental room were controlled for by using blackout curtains surrounding the testing area, ensuring that natural light from outside was not a factor. All subjects were tested in the same location under the same overhead lights in the testing space, and the luminance of our screen was constant.

## Results

**Figure 2:**
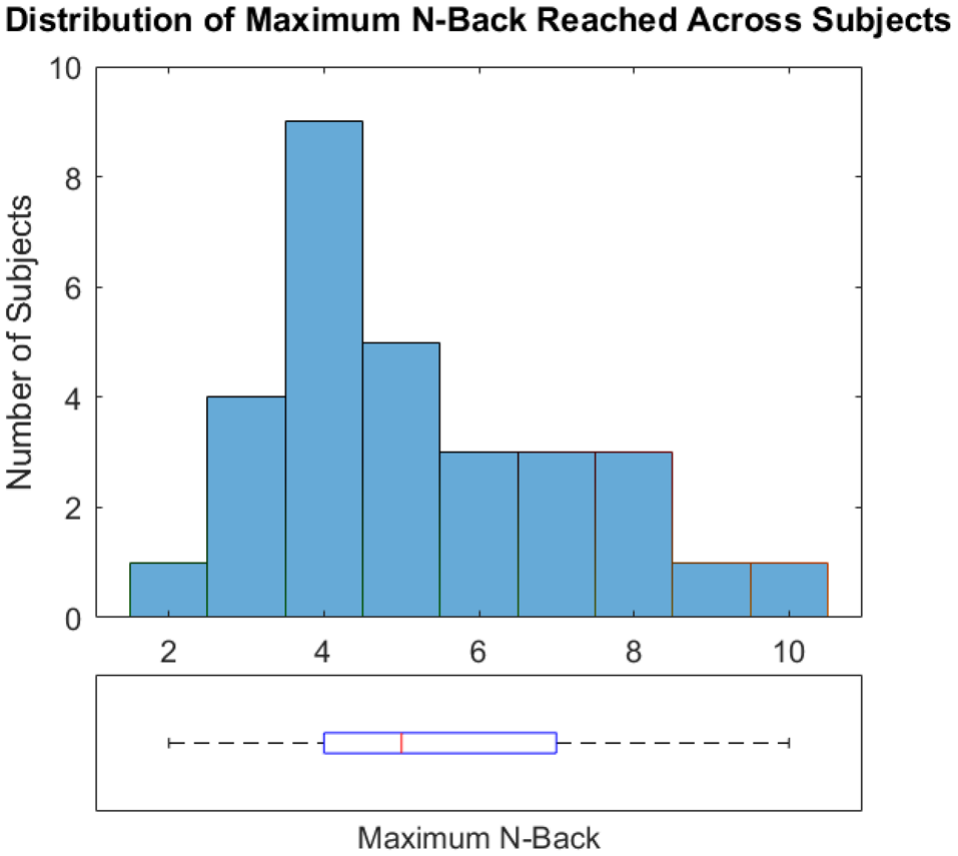
Distribution of maximum N-back achieved by each subject. Top plot represents a histogram of maximum N-back achieved by each participant. Bottom plot represents a box and whisker summary of the same data. The red line in the boxplot represents the median. The blue box represents the interquartile range (IQR) where the lower bound signifies the 25^th^ percentile (Q1) and the upper bound signifies the 75th percentile (Q3). The lower bound of the black line represents the minimum value, and the upper bound of the black line represents the maximum value.

In total, 1% of trials were missing due to Eyelink error (30 out of 3000 total trials). There were high levels of individual differences in performance on this task: the highest maximum N-back reached was 10 (1 participant), and the lowest maximum N-back reached was 2 (1 participant). The median N-back reached was 5, and the mode was 4 (Fig. 2). This wide distribution of maximum N-back achieved demonstrates the variability of subjects on this task, while simultaneously demonstrating that this adaptive task can be suited to a number of participants, regardless of overall ability on the cognitive load task.

**Figure 3:**
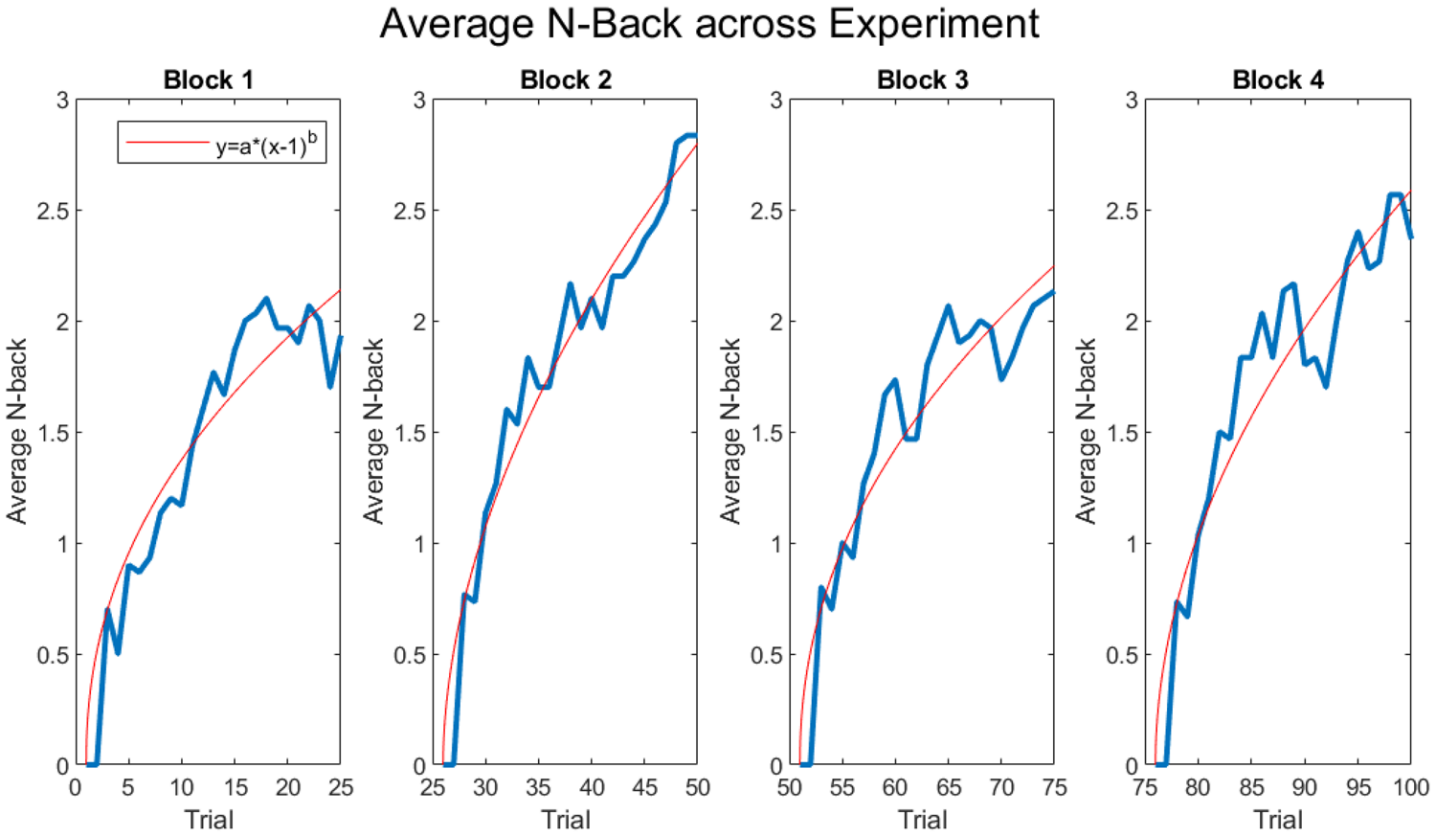
Mean N-Back across Experiment. Each sub-plot represents a block from the experiment, note N-Back resets to 0 at the start of each block. Means are computed as the average N-back from every subject at a given trial. Performance curves are fitted with a quadratic polynomial (*y* = a*(x−1)^b).

There were both slight learning and fatigue effects throughout the experiment, providing evidence that our task was successful in increasing cognitive load. We compared the rate of learning across each block by performing individual t-tests on the *b* value of our fit equation (*y* = a*(x−1)^b). We fit all 4 curves individually, found their average *a* value (0.5096), and set this as the constant *a*. By fitting all 4 blocks with an average constant, we were able to compare strictly the *b* value of each curve, or the rate of learning. Throughout each block there was a steady learning effect, and as the blocks continued, the rate of learning generally increased (Fig. 3). Compared to block 1, the rate of learning was faster in block 2 (t(29) = − 6.824, p < 0.001) and in block 4 (t(29) = − 4.276, p < 0.001), but not in block 3 (t(29) = − 1.140, p = 0.1318), demonstrating a possible fatigue effect that occurs just after the halfway point in the experiment. Learning is recovered in block 4, where the rate is significantly higher than in block 3 (t(29) = − 3.386, p = 0.001). The rate of learning was highest in block 2, where it was significantly faster than block 1 (t(29) = − 6.824, p < 0.001), block 3 (t(29) = − 6.082, p < 0.001), and block 4 (t(29) = − 2.094, p = 0.023).

### Response latency

**Figure 4:**
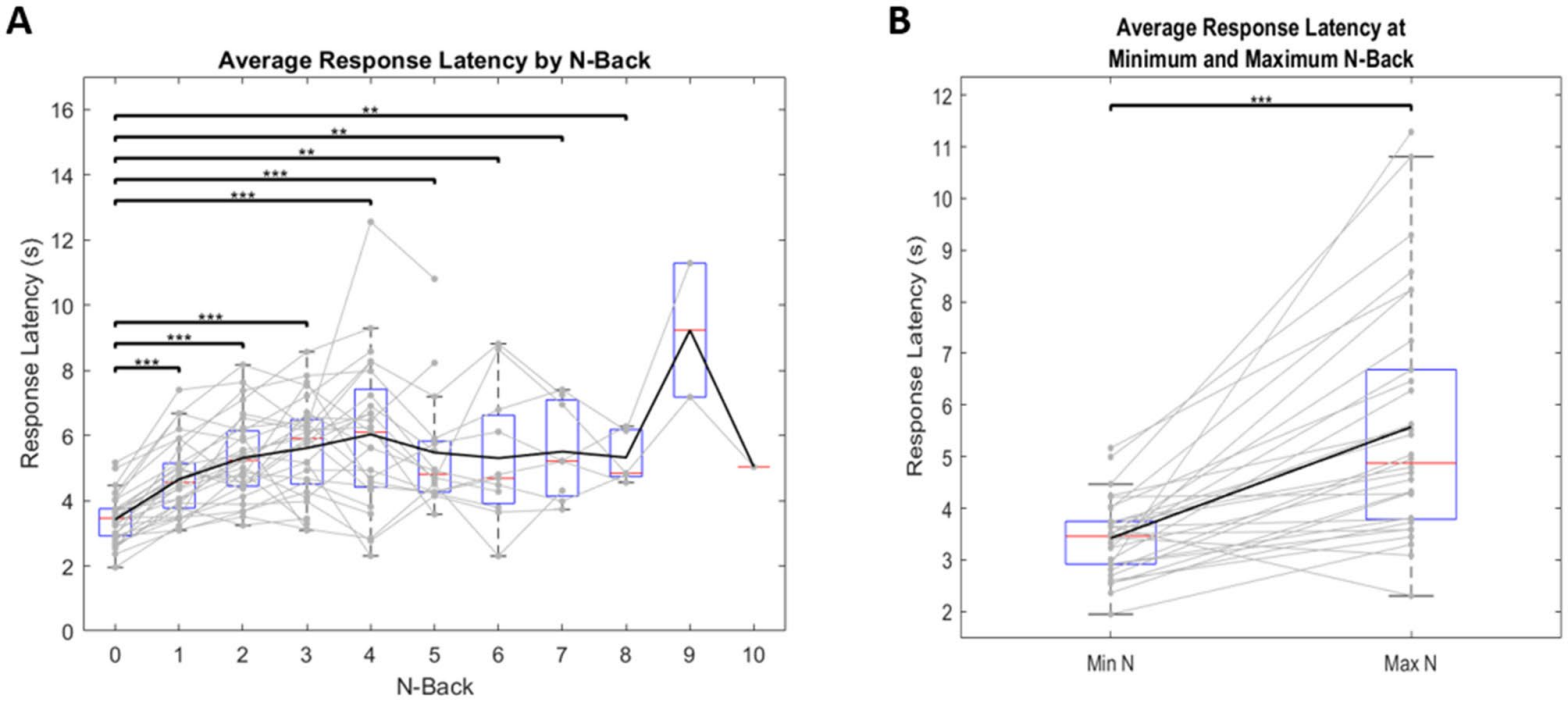
Response latency across N-back. Black line represents mean values. Solid gray lines represent individual subjects. Red lines represent median values. Blue boxes represent the IQR where each lower bound signifies Q1 and each upper bound signifies Q3. Lower and upper bounds of grey dotted lines represent minimum and maximum values, respectively. Points that lie outside these bounds are considered outliers. One star represents a significance value of p < 0.05, two stars represents a significance value of p < 0.01, and three stars represents a significance value of p < 0.001. (**A**) Demonstrates the average response times across each N-back. For clarity, only interactions against N = 0 are plotted here. (**B**) Demonstrates the average response latency at each subject’s minimum N-back (N = 0 for all subjects), compared to their maximum N-back (variable across subjects).

Because we had unequal sample sizes for each group, we ran conservative paired t-tests comparing average subject response times at each N-back. Replicating previous studies^26–28^, there was a significant increase in response latency concurrent with an increase in N-back. Specifically, an N-back of 0 was significantly faster than most other N-backs (Fig. 4A). This increase in reaction time plateaus around 5 s at an N-back of 4. Similarly, a paired t-test revealed that when comparing the minimum N-back achieved for each subject (N = 0 in all cases) or the “low-load” condition to the maximum N-back achieved (variable for all subjects) or the “high-load” condition, there was a significant increase in response time (t(29) = − 5.717, p < 0.001) (Fig. 4B). This suggests that our paradigm was successful in actively engaging working memory, as subjects demonstrated more difficulty in recalling the correct response as the N-back increased. This increase in response time is indicative of subjects having to work harder to search short term memory as difficulty of the task increases. Furthermore, when analyzing each subject individually, 26/30 subjects (86.7%) showed significant correlations (p < 0.05) between response latency and N-back. These results suggest that our paradigm successfully increases cognitive load and also adapts to individual differences in skill level on the task, and thus can easily accommodate the ability of different subjects.

### Pupilometry

**Figure 5:**
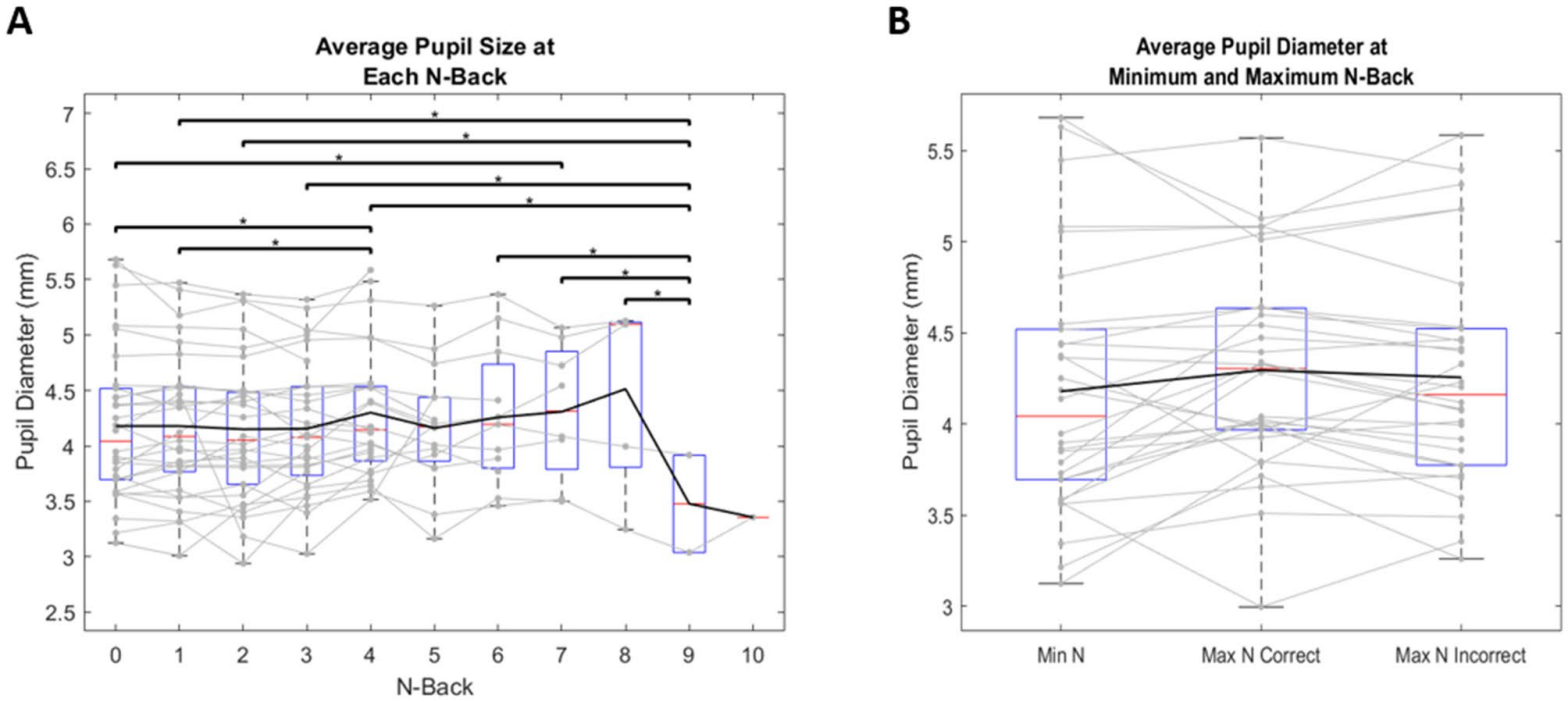
Mean pupil size as a function of N-back. Black line represents mean values. Solid gray lines represent individual subjects. Red lines represent median values. Blue boxes represent the IQR where each lower bound signifies Q1 and each upper bound signifies Q3. Lower and upper bounds of grey dotted lines represent minimum and maximum values, respectively. Points that lie outside these bounds are considered outliers. One star represents a significance value of p < 0.05, two stars represents a significance value of p < 0.01, and three stars represents a significance value of p < 0.001. (**A**) Pupil size increases gradually with increasing N-back, mainly at an N = 4 (most commonly reached maximum N-back), then constricts abruptly once N-back exceeds 8. (**B**) Average pupil size within subjects separated into minimum cognitive load (Min N) and maximum successful cognitive load (Max N Correct) and surpassed cognitive load (Max N Incorrect).

We measured pupil size using the reported average pupil diameter for each trial from the Eyelink. Pupil measurements were retrieved from the free-view portions of the experiment. Evidence suggests cognitive load can be measured through pupil diameter, where an increase in cognitive demand is associated with an increase in pupil size^31–34^. Our results replicate this finding, with a one-way ANOVA reporting a significant interaction of pupil size and N-back (F(10,2989) = 2.176, p = 0.017). Pupil size slightly increases as N-back increases, and then sharply drops off at an N-back of 9 or 10 (Fig. 5A), however there is a significant amount of inter-subject variability. Similarly, if we compare subject’s minimum cognitive load to their maximum successful cognitive load, we see a slight increase in pupil diameter, which then decreases once load is surpassed, demonstrated as incorrect trials at maximum load, however these interactions do not reach significance ((t(29) = − 1.671, p = 0.053) and (t(29) = 0.891, p = 0.190), respectively) (Fig. 5B). This trend is consistent with previous reports, which have shown that pupils dilate with the increasing demands of a working memory task, and then constrict again when cognitive load capacity has been surpassed^31^. This was a secondary analysis, and thus we did not compute the sample size required to power this analysis appropriately, however the trends we observe here are consistent with previous findings. Note that the distribution of luminance in our stimuli is non-uniform and the intensity of the pixels at each fixation may vary, depending on the subject’s fixation locations and the area of the image used to compute local intensity. The image sequences were randomized across subjects and sessions, so there is no relationship between N for a given subject and the stimuli that could account for these relationships.

### Fixations and saccades

**Figure 6:**
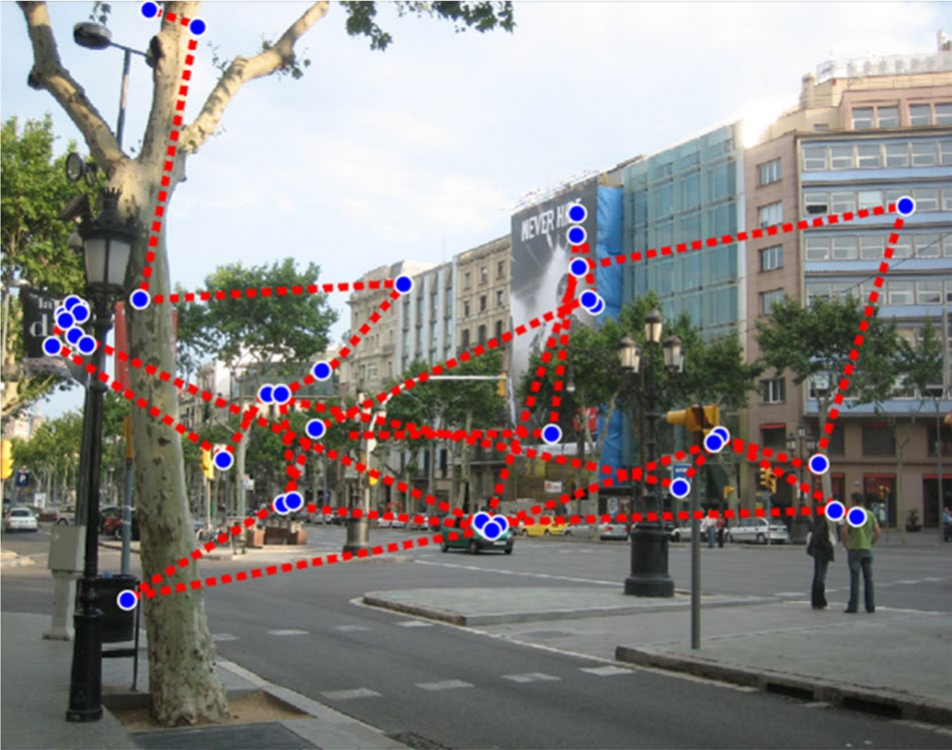
Example of a representative gaze-pattern from one subject. Blue points represent fixations, red dotted lines represent saccades. Image is from the LabelMe database^45^, made publicly available to the research community without restrictions.

We used the threshold criteria of the Eyelink 1000 to analyze the number of fixations and saccades, and durations of fixations and saccades. Standard settings on the Eyelink use a velocity threshold of 30°/s and an acceleration threshold of 8000°/s^2^ to determine the onset and offset of saccades (samples below these thresholds are considered to be fixational/microsaccadic eye movements). We only counted fixations or saccades occurring within the scene region, any events falling outside the image presented were discarded (amounting to a total of 1.59% data removal). Events for each trial were taken from one eye only: the eye used was determined by smoothing the position data of each eye and comparing the smoothed data to the original binocular data, and the eye with a smaller error was used. The total number of fixations and saccades that the Eyelink recorded during a trial were recorded, and the duration of fixations and saccades were the total cumulative time spent performing each type of event. Fixation and saccade data was retrieved from the free-view portions of the experiment. An example of a subject’s scan-path is presented in Fig. 6.

### Averages across subjects at each N-back

**Figure 7:**
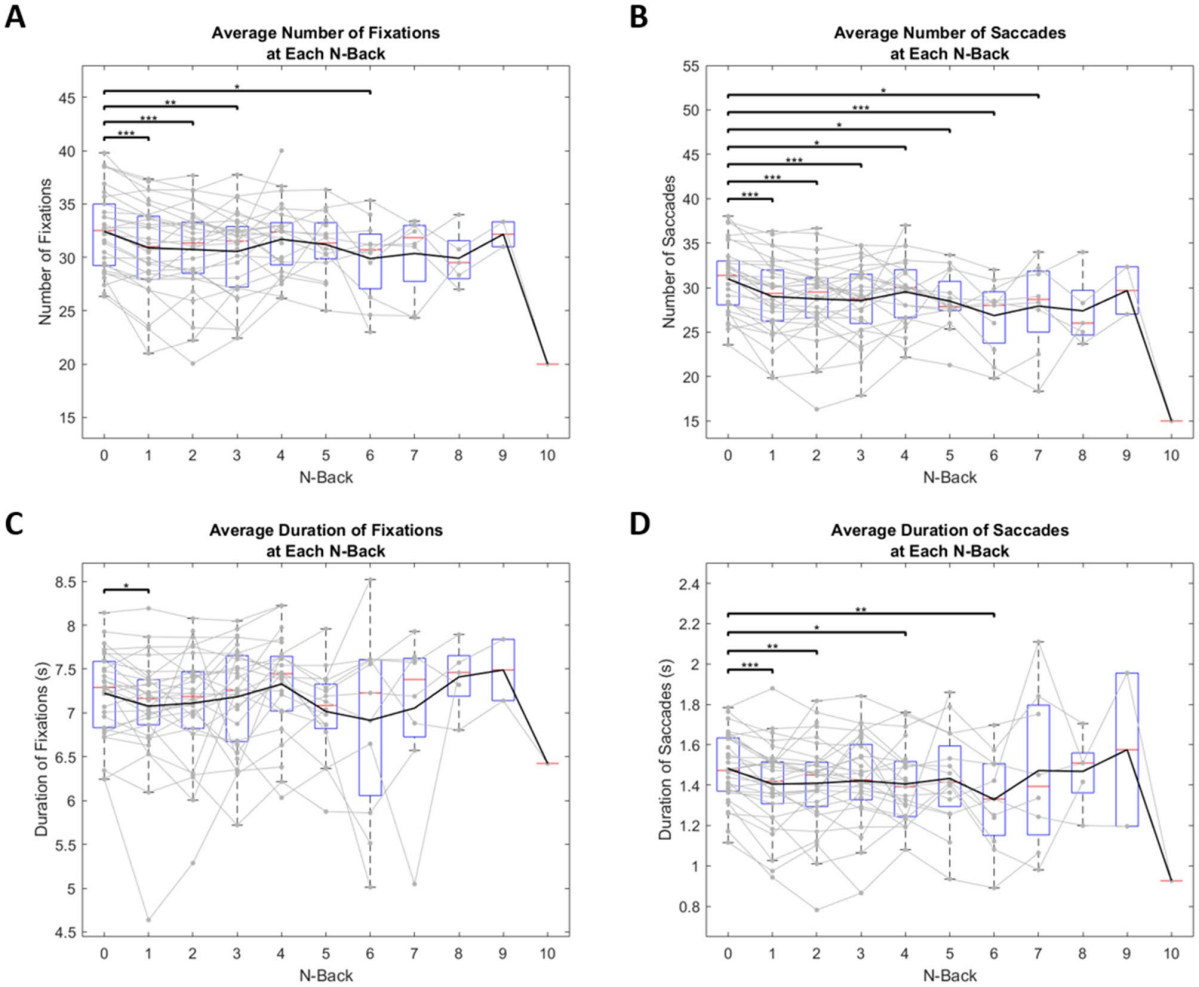
Average number (A&B) and duration (C&D) of fixations (A&C) and saccades (B&D) made by each subject at each N-back. Black line represents mean values. Solid gray lines represent individual subjects. Red lines represent median values. Blue boxes represent the IQR where each lower bound signifies Q1 and each upper bound signifies Q3. Lower and upper bounds of grey dotted lines represent minimum and maximum values, respectively. Points that lie outside these bounds are considered outliers. One star represents a significance value of p < 0.05, two stars represents a significance value of p < 0.01, and three stars represents a significance value of p < 0.001. For clarity, only interactions against N = 0 are plotted here. Number is represented as the total number of fixation or saccade events made during a trial. Duration is represented as the total cumulative sum of all fixation or saccade events during a trial.

We compared the number and duration of fixations and saccades made by each subject across all N-backs. We hypothesized that subjects who could achieve a higher N-back in our cognitive load task may use different oculomotor strategies than subjects who struggled to reach higher N-backs. We found this to be true, as there was a significant decrease in the number of oculomotor behaviors as N-back increased. We ran individual paired t-tests between each of the N-back groups. There was a significant decrease in the number of fixations (Fig. 7A), number of saccades (Fig. 7B), and duration of saccades (Fig. 7D). Mainly, there were significant differences between an N-back of 0 and most other N-backs. There was a small significant difference in the total duration of fixations from N = 0 to N = 1, but not at a wide variety of other Ns (Fig. 7C). If the number of fixations is decreasing but the overall time spent making fixations remains fairly constant, we can assume each individual fixation must be longer. This suggests that subjects who performed well in this task made fewer, longer fixations, to achieve success.

**Figure 8:**
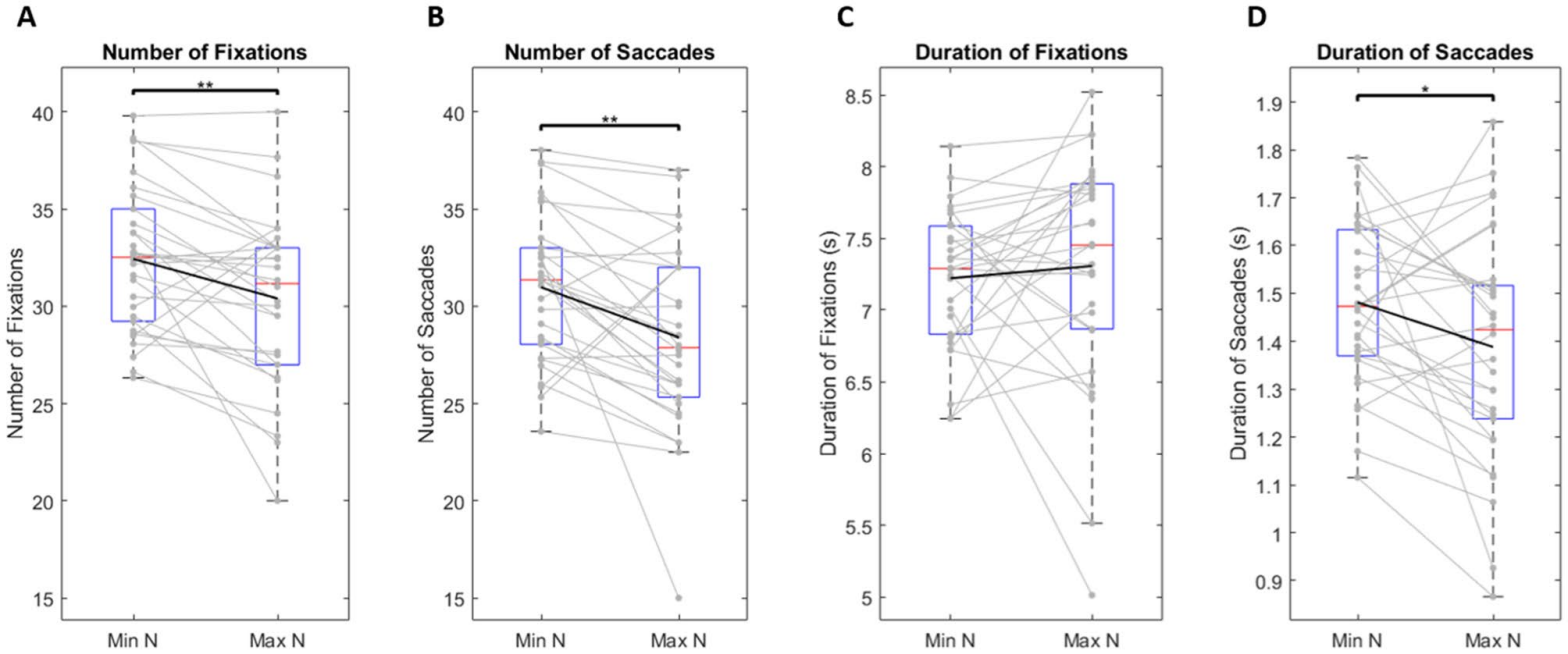
Average number (**A**,**B**) and duration (**C**,**D**) of fixations (**A**,**C**) and saccades (**B**,**D**) made by each subject at their minimum and maximum N-back achieved. Black line represents mean values. Solid gray lines represent individual subjects. Red lines represent median values. Blue boxes represent the IQR where each lower bound signifies Q1 and each upper bound signifies Q3. Lower and upper bounds of grey dotted lines represent minimum and maximum values, respectively. Points that lie outside these bounds are considered outliers. One star represents a significance value of p < 0.05, two stars represents a significance value of p < 0.01, and three stars represents a significance value of p < 0.001. Number is represented as the total number of fixation or saccade events made during a trial. Duration is represented as the total cumulative sum of all fixation or saccade events during a trial. Each plot demonstrates the average number or duration of fixations or saccades at each subject’s minimum N-back (N = 0 for all subjects), compared to their maximum N-back (variable across subjects).

We also looked at the number and duration of fixations and saccades made by each subject at their minimum N-back (lowest cognitive load), compared to their maximum N-back (highest cognitive load). We see the same trends in each of these conditions as well: the number of fixations (t(29) = 3.076, p < 0.01) (Fig. 8A), the number of saccades (t(29) = 3.268, p < 0.01) (Fig. 8B), and the duration of saccades (t(29) = 2.232, p < 0.05) (Fig. 8D) all decrease, while the duration of fixations (t(29) = -0.562, p = 0.579) (Fig. 8C) remains constant.

### Proportion of image looked at

**Figure 9:**
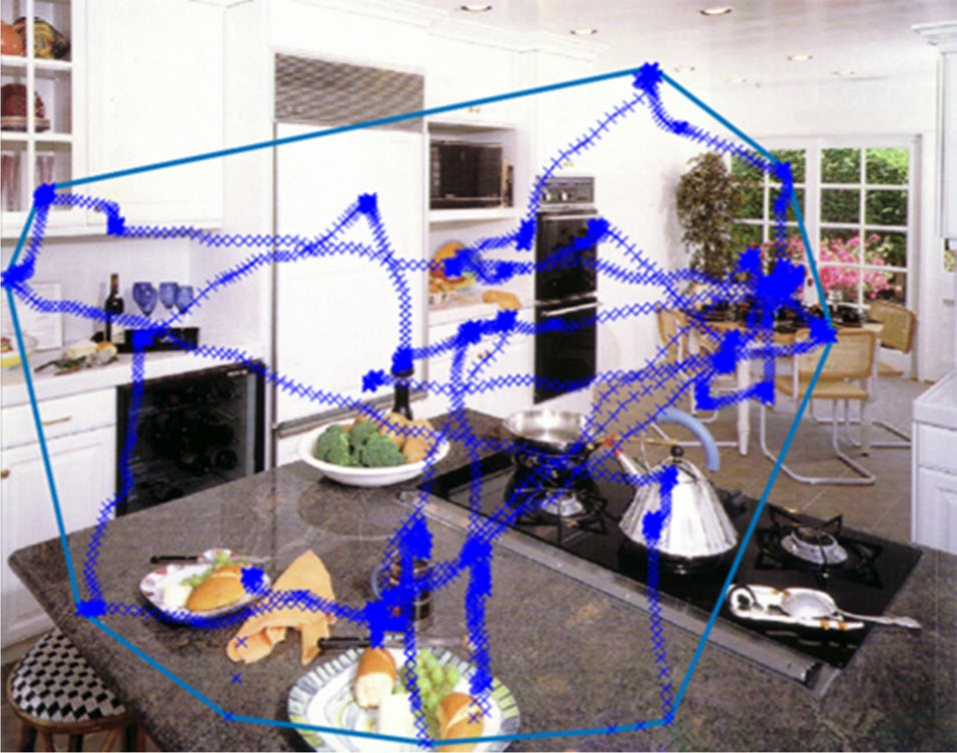
Percentage of image viewed analysis. Example of a subject’s raw gaze-data (blue x’s), overlaid with the convhull() polygon (teal lines). Area of the polygon is divided by the area of the image (not including gray stimulus background), to produce an approximate estimate of the percentage of the image viewed by the subject. Image is from the LabelMe Database^45^, made publicly available to the research community without restrictions.

Our analysis of the number and duration of fixations and saccades showed no relationships between task performance and high or low scoring subjects. We therefore looked at the proportion of each image viewed by each subject for each trial to examine whether there were any effects of the efficiency of eye movements and fixations. We used the convhull() function in Matlab to estimate the image area falling within the polygon defined by the farthest reaching positions recorded by the Eyelink (positions that fell outside of the image region were ignored). We used this as a measure of the approximate area of the image that was viewed by the subject. Values are represented as percentages, where the area of the image viewed was divided by the total size of the image (Fig. 9).

### Averages across subjects at each N-back

**Figure 10:**
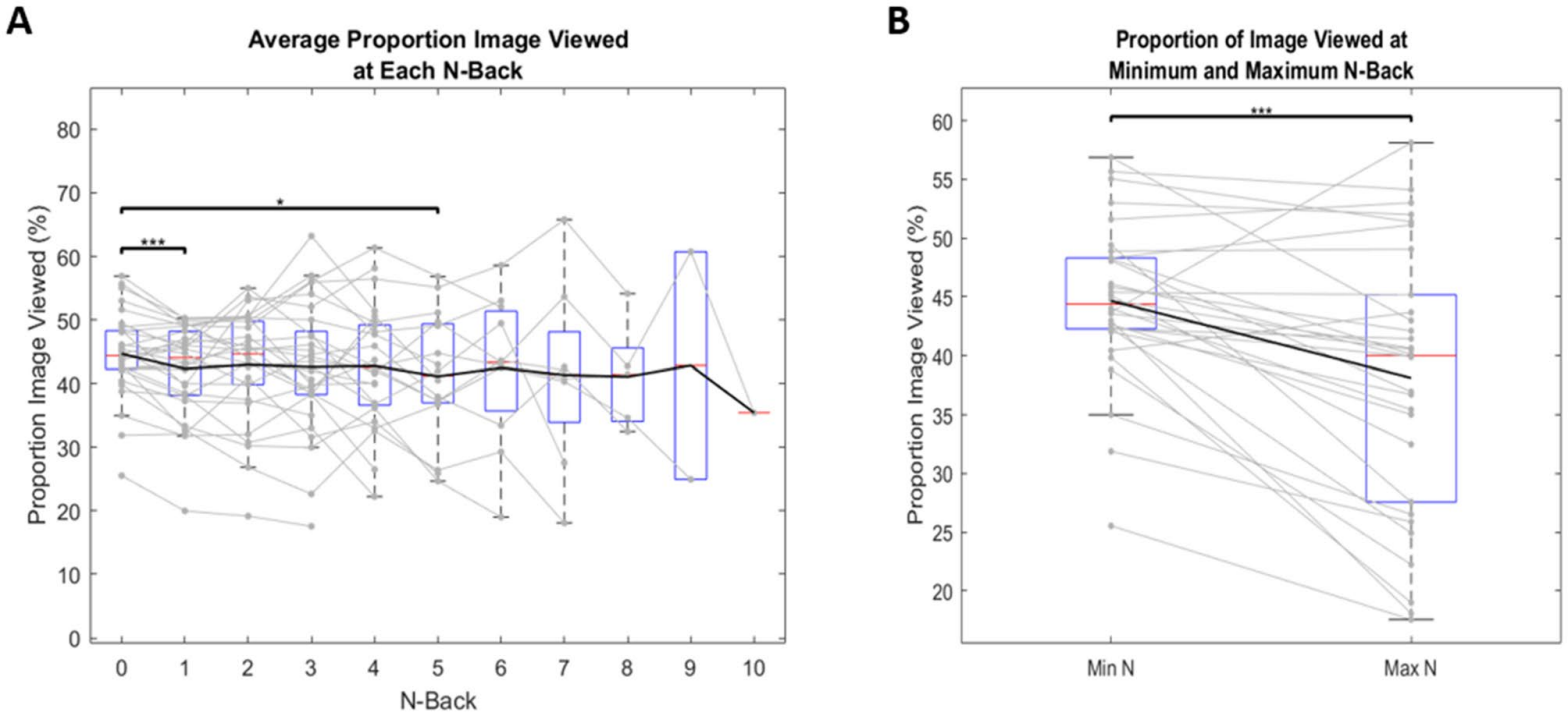
Proportion of image viewed. Black line represents mean values. Solid gray lines represent individual subjects. Red lines represent median values. Blue boxes represent the IQR where each lower bound signifies Q1 and each upper bound signifies Q3. Lower and upper bounds of grey dotted lines represent minimum and maximum values, respectively. Points that lie outside these bounds are considered outliers. One star represents a significance value of p < 0.05, two stars represents a significance value of p < 0.01, and three stars represents a significance value of p < 0.001. (**A**) Demonstrates average proportion of image viewed across each N-back. For clarity, only interactions against N = 0 are plotted here. (**B**) Demonstrates the average proportion of image viewed at each subject’s minimum N-back (N = 0 for all subjects), compared to their maximum N-back (variable across subjects).

Individual t-tests between each N-back found a significant decrease in the total proportion of image viewed as N-back increased, mainly between N = 0 and an N = 1 (Fig. 10A). Similarly, when comparing minimum and maximum cognitive load within subjects, there was a significant decrease in the proportion of image viewed at the maximum N-back achieved (Fig. 10B). This suggests that as cognitive load increases, subjects focus attention on a smaller sub-region of images.

## Discussion

We developed a novel adaptive paradigm to study how subjects view scenes under varying levels of cognitive demand. We found that our paradigm was successful in engaging working memory across various difficulties for individual subjects, as reflected in response latency and pupilometry analyses. Our paradigm demonstrates flexibility between subjects: the difficulty of the task is determined entirely by a subject’s ability to perform it. This allows the model to fit a variety of different participants with varying cognitive load capacities, while still being able to compare performance between and within subjects at different performance levels. A subject who can only reach an N-back of 2 still has a personalized low-load and high-load range that can be measured: N = 0 being low cognitive demand and N = 2 being high cognitive demand for this subject. Comparatively, a subject who can reach up to an N-back of 10 is also studied across their performance range, they still complete trials at low and high levels of cognitive load. In this way, the paradigm easily adapts to the subjective ability of individual participants. This feature potentially allows the paradigm to be deployed in special populations, an avenue we are currently investigating.

In our task, observers are required to free-view a sequence of natural images and identify objects from those images at a later stage. Results from Belke et al.^38^ demonstrate that a variety of different natural images can be presented in our task without the fear of perceptual load influencing oculomotor strategies. This provides assurance that any differences that we observed in oculomotor behaviors are due to the successful manipulation of cognitive load, rather than perceptual load. Luminance is controlled to some degree via random presentation of images, however varying luminance values both within and between our images may be a limitation of this study.

Any learning effects we witnessed were very small, with the average N-back per block only changing by about half an N at a time. This strengthens our claim that the differences in oculomotor behaviors witnessed were due to an increase in cognitive load, not because subjects were learning how to perform the task more efficiently.

When looking at the number and duration of fixations and saccades, we hypothesized that as N-back increased, the number of fixations and saccades would increase as subjects looked more exhaustively around the scene. An alternative hypothesis might state the number of fixations and saccades would decrease as subjects focused more steadily on significant portions of the scene. We found the latter hypothesis to be true: there was a significant decrease in the number of fixations and saccades, where the total duration of saccades decreased along with the number of events, while the total duration of fixations remained constant. This is consistent with oculomotor behaviors of cognitively impaired individuals, as children with Autism Spectrum Disorder (ASD) perform fewer fixations when processing social information^21^, children with Cerebral Visual Impairment (CVI) are severely deficient when scanning the environment, in some cases not scanning at all^41^, and adults with Alzheimer’s Disease (AD) demonstrate a decline in fixations with disease onset^42^.

These results suggest that increasing the demands of a cognitive load task affects oculomotor strategies, and different oculomotor behaviors may predict better performance on this task. These results support the assumption that oculomotor behavior differences between different neurological populations directly relate to attention and cognitive load.

Furthermore, there was a negative relationship between the proportion of the image viewed on average and performance in this task. Subjects who were more successful at this task overall did not fixate a higher overall area of each image, in fact the average proportion of the image viewed decreased as cognitive load was increased. These results, together with our oculomotor analysis, suggest that simply viewing “more” of an image does not necessarily improve performance. Searching out to the corners of each image does not predict better performance than focusing on a smaller, central area. Making fewer, longer fixations on more distinct areas of scenes correlates with higher success while performing tasks under high cognitive load.

Higher demands of cognitive load did affect the oculomotor behaviors between participants. As cognitive load increased, subjects made fewer, longer fixations. Because performance in this task is correlated with fewer oculomotor movements, we are interested in analyzing if the variability in success in this task may be reliant on scene context. Perhaps it isn’t the potential *amount* of information gathered during free-view, but rather the *context* of what was viewed. We are currently using semantic information of the fixated locations^15^ to examine whether success in this task correlates with salience-based viewing methods, or meaning-based ones.

Overall, this paradigm has great potential in measuring eye-movement data while controlling individualized cognitive load. Our pupilometry and performance data demonstrates that this task is successful in manipulating cognitive load while tailoring difficulty to the individual. Concurrently, our eyetracking data is consistent with the emerging idea that oculomotor behavior is a covert metric for cognitive load.

## References

[1] Henderson J (2003). Human gaze control during real-world scene perception. Trends Cogn. Sci..

[2] Rayner K (2009). The 35th sir Frederick Bartlett lecture: Eye movements and attention in reading, scene perception, and visual search. Q. J. Exp. Psychol..

[3] Buswell GT (1935). How People Look at Pictures: A Study of the Psychology and Perception in Art.

[4] Yarbus AL (1967). Eye Movements During Perception of Complex Objects.

[5] Andrews TJ, Coppola DM (1999). Idiosyncratic characteristics of saccadic eye movements when viewing different visual environments. Vision Res..

[6] Borji A, Sihite DN, Itti L (2013). Objects do not predict fixations better than early saliency: A re-analysis of Einhauser et al.’s data. J. Vis..

[7] Harel J, Koch C, Perona P (2007). Graph-based visual saliency. Adv. Neural Inf. Process. Syst..

[8] Itti L, Koch C (2001). Computational modelling of visual attention. Nat. Rev. Neurosci..

[9] Parkhurst D, Law K, Niebur E (2002). Modeling the role of salience in the allocation of overt visual attention. Vision Res..

[10] Henderson JM, Hayes TR, Peacock CE, Rehrig G (2019). Meaning and attentional guidance in scenes: A review of the meaning map approach. Vis. Switz..

[11] Hwang AD, Wang H-C, Pomplun M (2011). Semantic guidance of eye movements in real-world scenes. Vision Res..

[12] Nyström M, Holmqvist K (2008). Semantic override of low-level features in image viewing—both initially and overall. J. Eye Mov. Res..

[13] Onat S, Açık A, Schumann F, König P (2014). The contributions of image content and behavioral relevancy to overt attention. PLoS ONE.

[14] Rider AT, Coutrot A, Pellicano E, Dakin SC, Mareschal I (2018). Semantic content outweighs low-level saliency in determining children’s and adults’ fixation of movies. J. Exp. Child Psychol..

[15] Rose D, Bex P (2020). The linguistic analysis of scene semantics: LASS. Behav. Res. Methods.

[16] Stoll J, Thrun M, Nuthmann A, Einhäuser W (2015). Overt attention in natural scenes: Objects dominate features. Vision Res..

[17] Einhäuser W, Atzert C, Nuthmann A (2020). Fixation durations in natural scene viewing are guided by peripheral scene content. J. Vis..

[18] Nuthmann A (2017). Fixation durations in scene viewing: Modeling the effects of local image features, oculomotor parameters, and task. Psychon. Bull. Rev..

[19] Pedziwiatr MA, Kümmerer M, Wallis TSA, Bethge M, Teufel C (2021). Meaning maps and saliency models based on deep convolutional neural networks are insensitive to image meaning when predicting human fixations. Cognition.

[20] Ozeri-Rotstain A, Shachaf I, Farah R, Horowitz-Kraus T (2020). Relationship between eye-movement patterns, cognitive load, and reading ability in children with reading difficulties. J. Psycholinguist. Res..

[21] Howard PL, Zhang L, Benson V (2019). What can eye movements tell us about subtle cognitive processing differences in autism?. Vision.

[22] Wang S (2015). Atypical visual saliency in autism spectrum disorder quantified through model-based eye tracking. Neuron.

[23] Molitor RJ, Ko PC, Ally BA (2015). Eye movements in Alzheimer’s disease. J. Alzheimers Dis. JAD.

[24] Sweller J (1988). Cognitive load during problem solving: Effects on learning. Cogn. Sci..

[25] Kirchner WK (1958). Age differences in short-term retention of rapidly changing information. J. Exp. Psychol..

[26] Carlson S (1998). Distribution of cortical activation during visuospatial n-back tasks as revealed by functional magnetic resonance imaging. Cereb. Cortex.

[27] Jonides J (1997). Verbal working memory load affects regional brain activation as measured by PET. J. Cogn. Neurosci..

[28] Perlstein WM, Dixit NK, Carter CS, Noll DC, Cohen JD (2003). Prefrontal cortex dysfunction mediates deficits in working memory and prepotent responding in schizophrenia. Biol. Psychiatry.

[29] Braver TS (1996). A parametric study of prefrontal cortex involvement in human working memory. Neuroimage.

[30] Manoach DS (1997). Prefrontal cortex fMRI signal changes are correlated with working memory load. NeuroReport.

[31] Granholm E, Asarnow R, Sarkin A, Dykes K (1996). Pupillary responses index cognitive resource limitations. Psychophysiology.

[32] Kahneman D (1973). Attention and Effort.

[33] Klingner, J., Kumar, R. & Hanrahan, P. Measuring the task-evoked pupillary response with a remote eye tracker. in *Proceedings of the 2008 symposium on Eye tracking research & applications - ETRA ’08* 69 (ACM Press, 2008). 10.1145/1344471.1344489.

[34] Rafiqi, S. *et al.* PupilWare: towards pervasive cognitive load measurement using commodity devices. in *Proceedings of the 8th ACM International Conference on PErvasive Technologies Related to Assistive Environments - PETRA ’15* 1–8 (ACM Press, 2015). 10.1145/2769493.2769506.

[35] Stuyven E, Claeys K, Crevits L (2000). The effect of cognitive load on saccadic eye movements. Acta Psychol. (Amst.).

[36] Zagermann, J., Pfeil, U. & Reiterer, H. Measuring Cognitive Load using Eye Tracking Technology in Visual Computing. in *Proceedings of the Beyond Time and Errors on Novel Evaluation Methods for Visualization - BELIV ’16* 78–85 (ACM Press, 2016). 10.1145/2993901.2993908.

[37] Zagermann, J., Pfeil, U. & Reiterer, H. Studying Eye Movements as a Basis for Measuring Cognitive Load. in *Extended Abstracts of the 2018 CHI Conference on Human Factors in Computing Systems* 1–6 (ACM, 2018). 10.1145/3170427.3188628.

[38] Belke E, Humphreys GW, Watson DG, Meyer AS, Telling AL (2008). Top-down effects of semantic knowledge in visual search are modulated by cognitive but not perceptual load. Percept. Psychophys..

[39] Owen AM, McMillan KM, Laird AR, Bullmore E (2005). N-back working memory paradigm: A meta-analysis of normative functional neuroimaging studies. Hum. Brain Mapp..

[40] Meule A (2017). Reporting and interpreting working memory performance in n-back tasks. Front. Psychol..

[41] Salati R, Borgatti R, Giammari G, Jacobson L (2002). Oculomotor dysfunction in cerebral visual impairment following perinatal hypoxia. Dev. Med. Child Neurol..

[42] Mapstone M, Rösler A, Hays A, Gitelman DR, Weintraub S (2001). Dynamic allocation of attention in aging and Alzheimer disease: Uncoupling of the eye and mind. Arch. Neurol..

[43] Brainard DH (1997). The psychophysics toolbox. Spat. Vis..

[44] Cornelissen FW, Peters EM, Palmer J (2002). The Eyelink Toolbox: Eye tracking with MATLAB and the psychophysics toolbox. Behav. Res. Methods Instrum. Comput..

[45] Russell BC, Torralba A, Murphy KP, Freeman WT (2008). LabelMe: A database and web-based tool for image annotation. Int. J. Comput. Vis..

